# Management and Complications of Dexamethasone Intravitreal Implant Migration Into the Anterior Chamber

**DOI:** 10.1177/24741264221138166

**Published:** 2022-12-07

**Authors:** Devin M. Betsch, R. Rishi Gupta

**Affiliations:** 1 Department of Ophthalmology & Visual Sciences, QEII Health Sciences Centre, Halifax, NS, Canada

**Keywords:** dexamethasone intravitreal implant, implant migration, corneal transplant

## Abstract

**Purpose:**

To report patient demographics, the clinical course, and visual outcomes across a case series of patients who experienced dexamethasone (DEX) intravitreal implant (Ozurdex, Allergan, Inc) migration into the anterior chamber (AC), with a focus on the corneal transplantation rate.

**Methods:**

In this retrospective cross-sectional study, a chart review of all cases was performed. For numerical responses, means and SDs were calculated. Percentages and absolute numbers were used to report the proportion of patients who experienced various outcomes of interest.

**Results:**

The study comprised 32 cases. All cases occurred in pseudophakic eyes; of those, 8 (25.0%) had posterior chamber intraocular lenses in the capsular bag with no known capsular or zonular issues. The mean duration from DEX implant injection to detection of migration was 19.4 ± 14.5 days. The DEX implant was explanted in 21 patients (65.6%) and repositioned in the vitreous cavity or subconjunctival space in 6 patients (18.8%). Twelve patients (37.5%) ultimately required corneal transplantation.

**Conclusions:**

To our knowledge, this is the largest case series of DEX intravitreal implant migration into the AC compiled to date. Cases of migration occurred in individuals with no known history of significant prior zonule disruption. This potential complication should be discussed with all patients undergoing DEX implant injection, which may aid in earlier presentation and improved visual outcomes.

## Introduction

Dexamethasone (DEX) intravitreal implants (Ozurdex, Allergan, Inc) are a sustained drug-delivery system consisting of 700 µg of dexamethasone in poly(lactic-co-glycolic acid**)**. This implant can be injected into the vitreous cavity of the eye, permitting local release of the steroid at a low dose over several months. DEX implants are intended to stay suspended within the vitreous for the duration of that time. These devices can be effective in treating postoperative, inflammatory, and diabetic macular edema.^1^ The side effects associated with DEX implants are similar to those with other steroids and include cataracts and elevated intraocular pressure, which can be managed with surgical or pharmacological intervention. Other less commonly reported complications are retinal detachment, vitreous hemorrhage, and endophthalmitis.^1^

Over the past decade, there have been an increasing number of case reports describing the migration of DEX implants into the anterior chamber (AC).^2–13^ Some of these cases have been effectively managed with a combination of patient positioning, dilation, and gentle tapping on the eye, permitting repositioning of the implant back into the vitreous cavity.^3,5^ Others have described urgent surgical removal of the implant from the AC.^10,13^ The effect of these migrated implants on vision and corneal endothelium integrity is variable, ranging from minimal changes to nonresolving corneal edema. These corneal changes can progress despite removal of the implant, necessitating corneal surgery.^10,13^

Several individual case reports of migration have been published. The largest case series described 18 episodes across 15 patients.^13^ Our goal was to collect cases of DEX implant migration into the AC across multiple institutions to compile a large, comprehensive case series, with a focus on the proportion of patients who required corneal transplantation. We compare our dataset to the existing body of literature as well as highlight risk factors for migration so that ophthalmologists working with DEX implants may be better able to identify patients at increased risk of this potential complication.

## Methods

This study was performed in accordance with the principles outlined in the Declaration of Helsinki. Ethical approval for this study was obtained by the Nova Scotia Health Authority Research Ethics Board (approval number: 1026609). Informed consent was not sought for the present study as a waiver of consent was approved during the ethics board submission based on the possibility of a lack of a continued or existing relationship with the data holder at the time of the retrospective chart review.

This retrospective cross-sectional study comprised patients who experienced DEX implant migration into the AC. Consecutive cases from participating centers were included. If the case involved uneventful intravitreal injection of a DEX implant that migrated into the AC and there was enough information available to complete the circulated spreadsheet, the case was included. A consolidated document containing all cases from contributing sites was reviewed to ensure no duplicate cases were included. Data collected for each case included, but were not limited to, patient age, indication for DEX intravitreal implant injection, symptoms at the time of migration, duration from detection to treatment, type of intervention performed, and visual acuity (VA) before and after implant migration.

For numerical responses, means and SDs were calculated, and *t* tests were used to compare means, with significance defined as *P* < .05. Chi-square tests were used to compare distributions of categorical variables. Percentages and absolute numbers were used to report the proportion of patients who experienced the outcomes of interest, such as corneal transplantation, implant explantation, and conservative management.

## Results

A total of 32 cases of DEX implant migration into the AC were included. Thirty-three cases from 20 sites were submitted to the study, with 1 to 3 cases contributed by each site. One case was excluded as the DEX implant was erroneously injected into the lens and did not truly migrate from the vitreous cavity to the AC.

Table 1 shows the patients’ characteristics. The mean age of the patients was 66.8 ± 12.5 years; most were men. The most common indications for DEX injection were macular edema secondary to uveitis and postoperative inflammation. The ocular history was significant for previous pars plana vitrectomy (PPV), trauma, and neodymium:YAG capsulotomy

**Table 1.** Baseline Characteristics of 32 Patients Who Had DEX Intravitreal Implant Migration Into the Anterior Chamber.

Characteristic	Number (%)
Sex	
Male	28 (87.5)
Female	4 (12.5)
Involved eye	
Right	18 (56.3)
Left	14 (43.7)
DEX implant indication	
Uveitis	11 (34.4)
Postop inflammation	11 (34.4)
Diabetes	5 (15.6)
Retinal vein occlusion	4 (12.5)
Hypotony	1 (3.1)
IOL status	
PCIOL in capsular bag	13 (40.6)
Scleral-fixated IOL	9 (28.1)
PCIOL in sulcus	6 (18.8)
ACIOL	4 (12.5)
Ocular history	
Trauma	5 (15.6)
Previous PPV	24 (75.0)
Previous Nd:YAG capsulotomy	2 (6.3)

Abbreviations: ACIOL, anterior chamber intraocular lens; DEX, dexamethasone; IOL, intraocular lens; Nd:YAG, neodymium:YAG; Number, absolute number; PCIOL, posterior chamber intraocular lens; PPV, pars plana vitrectomy.

All 32 cases occurred in pseudophakic eyes (Table 1). Of note, 8 patients (25.0%) had a posterior chamber intraocular lens (PCIOL) in the capsular bag with no known capsular trauma or zonular disruption, although 6 of these patients had a previous PPV and 1 had a history of uneventful Nd:YAG capsulotomy. Of the 9 patients with a scleral-fixated IOL, 3 (33.0%) did not have a capsular bag and 2 (22.2%) had documented zonular dehiscence; in 4 cases (44.4%), the status of the capsular bag/zonular fibers was not explicitly mentioned.

Table 2 shows the features and management of patients with DEX intravitreal implant migration into the AC. The majority of patients presented with blurred vision and had corneal edema on initial examination. In the 2 patients who were asymptomatic on presentation, the implant migration was discovered on regular follow-up. The mean time from DEX implant injection to detection of migration was 19.4 ± 14.5 days.

**Table 2.** Presenting Features and Management of Patients With Dexamethasone Intravitreal Implant Migration Into the Anterior Chamber.

Characteristic	Number (%)
Sign/symptom at presentation	
Blurred vision	29 (90.6)
Pain	10 (31.3)
White line noticed^a^	3 (9.4)
Asymptomatic	2 (6.3)
Corneal edema	27 (84.4)
Management	
Explantation	21 (65.6)
Surgical repositioning	
Vitreous cavity	4 (12.5)
Subconjunctival	2 (6.3)
Dilation and positioning	1 (3.1)
Observation	4 (12.5)

Abbreviation: Number, absolute number.

a These patients noticed a “white line” in the anterior chamber on self-examination in a mirror.

Twenty-one patients ultimately had DEX implant explantation, and 6 had their implants surgically repositioned, either in the vitreous cavity or subconjunctival space. Four patients were observed and monitored until the implant dissolved; of those, 2 ultimately required corneal transplantation. One patient was treated conservatively with pupil dilation and positioning alone and did not require corneal transplantation. In 3 patients, the implant re-migrated into the AC after initial repositioning. One of these patients went on to have surgical explantation of the DEX implant, and 2 underwent surgical repositioning procedures (Table 2).

Twelve patients (37.5%) required corneal transplantation for nonresolving corneal edema, and 2 (6.3%) required multiple transplants as a result of graft failure. Two patients (6.3%) requiring corneal transplantation had a history of prior corneal transplantation in the same eye for reasons unrelated to DEX implant migration. The time between management of the migrated implant to the decision to pursue corneal transplantation ranged from 1 to 16 months.

Of the patients who required corneal transplantation, the most common indication for initial DEX implant injection was postoperative cystoid macular edema (6/12 [50.0%]), followed by uveitis (3/12 [25.0%]). One person each had hypotony, branch retinal vein occlusion with macular edema, and diabetic macular edema as their underlying diagnosis. Of the 2 patients who required multiple corneal transplants, 1 had uveitis and the other had hypotony.

Table 3 shows the statistical likelihood of outcomes of interest in patients with DEX implant migration into the AC. Of those who had surgical intervention, the time from symptom onset to surgery was statistically significantly longer in patients who ultimately required corneal transplantation than in those who did not (*P* = .007). Those who required corneal transplantation were statistically significantly more likely than those who did not have transplantation to have a final Snellen VA of 20/200 or worse at their most recent follow-up (*P* = .02).

**Table 3.** Statistical Likelihood of Outcomes of Interest in Patients With Dexamethasone Implant Migration Into the Anterior Chamber.

Parameter compared	Value
Days from injection to migration detection	
Corneal transplant group	15.7
No corneal transplant group	21.6
Days from symptom onset to surgery^a^	
Corneal transplant group	7.4
No corneal transplant group	2.4
Final VA ≤ 20/200 (%)^a^	
Corneal transplant group	66.6
No corneal transplant group	25.0
Likelihood of corneal transplant (%)	
Surgical group	34.6
No surgery group	50.0
Likelihood of corneal transplant (%)	
Implant removed group	33.3
Implant not removed group	45.5

Abbreviation: VA, visual acuity.

a Statistically significant difference between groups (*P* < .05).

The time from DEX implant injection to detection of migration was not significantly different between the group that required corneal transplantation and the group that did not (*P* = .283). There was no statistically significant difference in the corneal transplantation rate among those who were initially managed without surgery and those who had surgery (*P* = .48) or in those who had the DEX implant left in the eye and those whose implant was explanted (*P* = .50) (Table 3).

Sixteen cases of migration (50%) occurred with first DEX intravitreal implant injection. Two cases (6.3%) occurred in eyes with more than 10 previous uncomplicated DEX implant injections. Only 2 patients (6.3%) have received repeat treatment with DEX intravitreal implants after the injection complicated by AC migration.

## Conclusions

To our knowledge, this is the largest case series of DEX implant migration into the AC to date. A case series published by Khurana et al^13^ described 18 episodes of DEX implant migration in 15 patients. Of those, 6 (40.0%) required corneal transplantation, which is fairly consistent with the 37.5% transplantation rate in our case series.

PPV as a risk factor for intravitreal implant migration has been well documented and is thought to be caused by a lack of scaffolding or support for the implant, allowing more opportunity for anterior egress. All 15 patients in the study by Khurana et al^13^ had a previous PPV,^13^ as was the case in several other previously published case reports.^2,11,12^ Compounded risk occurs in patients who are concurrently aphakic.^2,13,14^ However, only 75% of our cases had a history of PPV, and none was aphakic. This suggests that although previous vitrectomy and aphakia increase the likelihood of DEX implant migration, patients lacking one or both of these risk factors should be counseled on the possibility of implant migration.

All patients in the study by Khurana et al had a disrupted capsular bag, zonular dialysis, or trauma, similar to several other reported cases that describe DEX implant migration in the setting of documented posterior capsule rupture.^11,13,15,16^ There have been published cases of implant migration in patients with intact lens capsules and zonular stability, although with patent iridectomies as the suspected route of entry into the AC.^3^ To our knowledge, there are only 4 published cases of AC migration in patients who were pseudophakic but had no documented capsule or zonular deficiencies.^5,6,10,12^ We had several patients with a PCIOL in the capsular bag and no documented trauma, surgical complications, zonular fiber disruption, or iridectomies. This makes it difficult to anticipate those who might experience this potentially vision-threatening complication, and warrants widespread and comprehensive counseling on this risk before DEX implant injection.

Various management options were used across the cases we collected, including monitoring for absorption, dilation and positioning, tapping on the globe, minor procedures using needles or a balanced salt solution for posterior repositioning, and a wide array of techniques for surgical removal. Sixty-seven percent of those in the study by Khurana et al^13^ had the migrated DEX implant removed, which is similar to the 65.6% rate in our study. Observation or conservative management alone was effective in mitigating the need for corneal transplantation in only 3 of our patients.

Only three patients in our study were documented as noticing a “white line” in their eye on gross examination (looking in a mirror), while all patients would have had this finding to some degree. Before implant injection, it may be helpful to show patients what a DEX implant looks like, provide clear visuals of the implant in the AC, and highlight this as an abnormal finding that warrants urgent ophthalmologic assessment. Discussion about the importance of symptoms associated with migration, such as decreased vision, redness, and pain, is also needed given that patients with a longer duration between symptom onset and surgical intervention were more likely to require corneal transplantation in the current study. Improved patient education can lead to better outcomes, as demonstrated in prior studies that have shown those undergoing surgical removal of the implant have a reduced likelihood of permanent corneal edema with earlier intervention.^13^

We acknowledge that not all cases of implant migration present for medical attention, given that some patients could remain asymptomatic and the migration might spontaneously resolve. Therefore, this study might underestimate the true incidence of DEX implant migration into the AC.

In conclusion, our large case series highlights the variety of patients who may present with DEX implant migration into the AC. Although previous vitrectomy, zonular or capsular bag disruption, aphakia, and iridectomy increase risk and provide a route for entry into the AC, it is not altogether uncommon for this complication to occur in patients with none of these risk factors. This potential complication, as well as its associated signs and symptoms, should be discussed with all patients having DEX intravitreal implant injection; such discussion might aid in earlier presentation and improved visual outcomes.

## References

[1] Rajesh B Zarranz-Ventura J Fung AT , et al. Safety of 6000 intravitreal dexamethasone implants. Br J Ophthalmol. 2020;104(1):39-46. 31040132 10.1136/bjophthalmol-2019-313991

[2] Bansal R Bansal P Kulkarni P Gupta V Sharma A Gupta A. Wandering Ozurdex^®^ implant. J Ophthalmic Inflamm Infect. 2012;2(1):1-5. 21960148 10.1007/s12348-011-0042-x PMC3302995

[3] Eadie JA Lesser R Capone A Jr. Migration of Ozurdex implant into the anterior chamber. Retin Cases Brief Rep. 2012;6(3):269-270. 25389729 10.1097/ICB.0b013e3182258b08

[4] Stepanov A Codenotti M Ramoni A , et al. Anterior chamber migration of dexamethasone intravitreal implant (Ozurdex^®^) through basal iridectomy (Ando) in a pseudophakic patient. Eur J Ophthalmol. 2016;26(3):e52-e54. 10.5301/ejo.5000715 26615950

[5] Collet B. Management of Ozurdex in the anterior chamber. JAMA Ophthalmol. 2013;131(12):1651-1652. 10.1001/jamaophthalmol.2013.5610 24337563

[6] Daudin JB Brézin AP. A white line in the anterior chamber. JAMA Ophthalmol. 2013;131(3):398. 23494046 10.1001/jamaophthalmol.2013.592

[7] Nguyen TL Wolfensberger TJ. Management of anterior chamber dislocation of a dexamethasone implant. Klin Monbl Augenheilkd. 2019;236(4):412-414. 30616285 10.1055/a-0808-1847

[8] Bernal L Estévez B. Corneal toxicity after Ozurdex^®^ migration into anterior chamber. Arch Soc Esp Oftalmol. 2016;91(6):292-294. 26922138 10.1016/j.oftal.2016.01.008

[9] Sborgia G Niro A D’Oria F , et al. Surgical management of complications after dexamethasone implant. Case Rep Ophthalmol Med. 2020;2020:4837689. 32095301 10.1155/2020/4837689 PMC7035535

[10] Kang H Lee MW Byeon SH Koh HJ Lee SC Kim M. The clinical outcomes of surgical management of anterior chamber migration of a dexamethasone implant (Ozurdex^®^). Graefes Arch Clin Exp Ophthalmol. 2017;255(9):1819-1825. 28601910 10.1007/s00417-017-3705-y

[11] Röck D Bartz-Schmidt KU Röck T. Risk factors for and management of anterior chamber intravitreal dexamethasone implant migration. BMC Ophthalmol. 2019;19(1):120. 31138164 10.1186/s12886-019-1122-1 PMC6537356

[12] Kayıkcıoğlu Ö Doğruya S Sarıgül C Mayalı H Kurt E . Anterior chamber migration of Ozurdex implants. Turk J Ophthalmol. 2020;50(2):115-122. 32367704 10.4274/tjo.galenos.2019.43778 PMC7204895

[13] Khurana RN Appa SN McCannel CA , et al. Dexamethasone implant anterior chamber migration: risk factors, complications, and management strategies. Ophthalmology. 2014;121(1):67-71. 23890421 10.1016/j.ophtha.2013.06.033

[14] Majumder PD Palkar AH Pathare N Biswas J. Anterior chamber migration of a sustained-release dexamethasone intravitreal implant: a case report and review of literature. Oman J Ophthalmol. 2019;12(2):133-137. 31198303 10.4103/ojo.OJO_5_2018 PMC6561047

[15] Pardo-López D Francés-Muñoz E Gallego-Pinazo R Díaz-Llopis M. Anterior chamber migration of dexametasone intravitreal implant (Ozurdex^®^). Graefes Arch Clin Exp Ophthalmol. 2012;250(11):1703-1704. 21861084 10.1007/s00417-011-1802-x

[16] Gullapalli VK DiLoreto DA. Migration of intravitreal dexamethasone implant to anterior chamber. Retin Cases Brief Rep. 2013;7(1):111-113. 25390539 10.1097/ICB.0b013e31826f08b0

